# Application of Multiphoton Microscopy in Dermatological Studies: a Mini-Review

**DOI:** 10.1142/S1793545813300103

**Published:** 2014-01-03

**Authors:** Elijah Yew, Christopher Rowlands, Peter T. C. So

**Affiliations:** * Singapore-MIT Alliance for Research and Technology (SMART), 1 CREATE Way CREATE Tower, Singapore 138602; † Department of Biological Engineering Massachusetts Institute of Technology 77 Massachusetts Ave, Cambridge MA 02139, USA; ‡ Department of Mechanical Engineering Massachusetts Institute of Technology 77 Massachusetts Ave, Cambridge MA 02139, USA; § GR Harrison Spectroscopy Laboratory 77 Massachusetts Ave, Cambridge MA 02139, USA

**Keywords:** Multiphoton microscopy, skin, cancer, aging and regeneration

## Abstract

This review summarizes the historical and more recent developments of multiphoton microscopy, as applied to dermatology. Multiphoton microscopy offers several advantages over competing microscopy techniques: there is an inherent axial sectioning, penetration depths that compete well with confocal microscopy on account of the use of near-infrared light, and many two-photon contrast mechanisms, such as second-harmonic generation, have no analogue in one-photon microscopy. While the penetration depths of photons into tissue are typically limited on the order of hundreds of microns, this is of less concern in dermatology, as the skin is thin and readily accessible. As a result, multiphoton microscopy in dermatology has generated a great deal of interest, much of which is summarized here. The review covers the interaction of light and tissue, as well as the various considerations that must be made when designing an instrument. The state of multiphoton microscopy in imaging skin cancer and various other diseases is also discussed, along with the investigation of aging and regeneration phenomena, and finally, the use of multiphoton microscopy to analyze the transdermal transport of drugs, cosmetics and other agents is summarized. The review concludes with a look at potential future research directions, especially those that are necessary to push these techniques into widespread clinical acceptance.

## 1. Introduction

The skin is the largest and most easily accessible organ in the body, and consequently, many optical methods are first developed for dermatological studies. The skin is also comparatively thin, having thickness compatible with ballistic penetration depth of light in tissues: on the order of hundreds of microns.^1^ These factors contributed to the early adoption of multiphoton microscopy to study skin physiology^2^ and the subsequent application of multiphoton microscopy to the diagnosis of skin cancer and other diseases, the monitoring of skin aging and regeneration processes, and transport studies of chemicals, drugs and nanoparticles through the skin, with implications in drug delivery, cosmetics and sun protection.

There are other excellent reviews of this field and readers looking for another perspective are invited to pursue these further. Previous reviews include an extensive review by Schenke-Layland *et al*. that summarized the field up to 2006^3^ and a shorter review by Lin *et al*. with a more clinical perspective.^1^ More recently, Wang *et al*. published a review focused primarily on ophthalmology but with a substantial section on skin.^4^ In another work, König *et al*. discussed the use of multiphoton imaging in drug delivery research, with emphasis on the treatment of photoaging.^5^ The use of multi-photon fluorescence lifetime imaging microscopy (FLIM) for skin imaging has attracted recent interest, with reviews by Cicchi and Pavone,^6^ Seidenari *et al*.,^7^ and König.^8^ Campagnola and Dong have summarized the use of second-harmonic generation in disease diagnosis, including its use in dermatology.^9^ Finally, Perry *et al*. have published a review on the use of multiphoton imaging in cancer research which contains a lengthy discussion on skin cancers.^10^

While many researchers have contributed to this field, it is easy to recognize the works of König and his co-workers as being singularly substantial. König and co-workers have contributed to almost every aspect of dermatological research based on multiphoton microscopy. More importantly, this group was the first to successfully commercialize multiphoton dermatological instrumentation that is compatible with clinical use. Their work has established the safety and efficacy of multiphoton imaging in the skin.^11^ They have further obtained regulatory approval for the clinical use of these instruments in the European Union and several other countries. The availability of commercial instruments has significantly increased the routine use of multiphoton microscopic imaging by clinicians; progress in this area has been summarized in several articles.^12–16^

This review will first focus on skin physiology and its optical properties. The historical development of multiphoton techniques for skin imaging will be briefly described. The focus of this review will then shift to cover the different dermatological diseases and phenomena that can be studied using multiphoton microscopy, and will close with a discussion on the future directions within this field.

## 2. Optical Considerations in Multiphoton Microscope Skin Imaging

The physiology and optical properties of the skin have been studied extensively. Knowledge of the optical properties of the skin allows the rational design of multiphoton microscopy technologies for dermal imaging. In this section, we will provide a brief overview of skin physiology with an emphasis on its optical properties. A brief history of the development of multiphoton microscope technol­ogies for skin imaging will also be provided.

### 2.1. Skin physiology and optical properties

Physiologically, skin may appear to be simple but is actually a complex organ consisting of two structurally distinct regions.^17^ The outer region, known as the epidermis, consists of stratified cells and is relatively thin, on the order of 100-150 *μ*m. The exterior of the epidermis is the *stratum corneum,* consisting of five or six layers of cornified dead cells that are in the process of being sloughed off. The next two strata, the stratum granulosum and the stratum spinosum, each consist of several layers of living keratinocytes. The keratinocytes in the stra­tum spinosum tend to be polyhedral in shape. The bottom stratum of the epidermal region is the basal cell layer, where the cells are in the process of pro­liferating and differentiating into new cells that migrate toward the surface. Four types of cells are located within the living epidermis. The majority are keratinocytes, with the few remaining percent being dendritic cells: Langerhans cells, melanocytes and Merkel cells. Melanocytes produce the pigment melanin which is formed into vesicles called melanosomes. Melanin is not a homogenous chemical species but can be roughly classified as either eumelanin or pheomelanin, each with distinct spectroscopic properties. Eventually the melanosomes are transferred from the melanocytes into the basal epithelial cells. The epidermis also contains other physiological structures, including free nerve endings, hair, sweat and apocrine glands.

The dermal region is located under the epidermis. It is important to note that the epidermal–dermal junction is not a flat surface, but has a characteristic, undulating reteridge structure. The dermis primarily consists of extracellular matrix tissue, including collagen and elastin fibers, and a sparse population of cells including fibroblasts, macrophages and adipocytes. Within the dermis, there are also many important functional organs, including hair follicles, sweat, sebaceous and apocrine glands, nerve fibers and their receptors. The hair follicle structure is particularly important as recent studies have shown that the skin stem cells are located in niches close to the base of the hair follicle; these stem cells are required for the repair of the skin after injury. The vasculature and the lymphatic vessels can also be found within the dermis. The capillary loops of the vasculature extending into the tips of the reteridge supply oxygen to the living cells both in the dermis and the epidermis. The thickness of the dermis ranges from hundreds of micrometers to millimeters, with the actual thickness depending on the location on the body and the species of the organism.

While skin structure and biochemistry can be studied with multiphoton microscopy using extrinsic contrast agents such as organic fluorophores or genetically expressible fluorescent proteins, it is important to note that many skin components can be imaged based on endogenous contrast. Keratinocytes can be visualized based on fluorescence from reduced pyridine nucleotides and oxidized flavin proteins. Both flavin proteins and NAD(P)H are localized in the cellular mitochondria, but NAD(P) H is also present in the rest of the cytosol. Importantly, cellular metabolism can be noninvasively monitored by redox fluorometry.^18,19^ Endogenous fluorescence imaging of the structural protein keratin that is abundant in the *stratum corneum* has also been reported.^20^ In the dermis, collagen and elastin are also observable based on their fluorescence. However, several isoforms of collagen, including type I, often produce a higher second harmonic generation (SHG) signal due to their noncentrosymmetric molecular structure and crystalline organization. The combination of the SHG signal with polarization-resolved imaging can further provide information on the elements of the *χ^2^* susceptibility matrix, which in turn can provide more detailed information regarding the molecular level organization of the collagen fibrils, such as their chirality. Because of the importance of melanoma as a disease, melanin has also been an im­portant target for multiphoton imaging. A recent study^21^ based on pump-probe transient absorption imaging has demonstrated the distinction between pheomelanin and eumelanin; their relative abun­dances has been postulated to be an oncogenic factor.^22^ Other reports suggest that melanin can be imaged based on a stepwise-multiphoton excitation process.^23^

Multiphoton imaging of the skin is complicated by its stratified structure, since the different structural layers have very distinct refractive indices, as studied by Tearney and co-workers.^24^ As a result of refractive index variation, the actual change in the focal depth of focused light differs from that expected by the physical translation of the speci­men, and this difference can be measured quantitatively using optical coherence tomography. They found that the refractive indices in *stratum corneum,* epidermis and dermis have values of 1.51, 1.34 and 1.41, respectively. Effectively, the *stratum corneum* has reflective index close to oil, while the epidermis has index close to water and the dermis lies in between. For multiphoton imaging, this multi-layered structure with different indices of refraction causes spherical aberration and distorts the excitation focus, resulting in signal loss and a reduction in image resolution. In other more hom­ogenous tissues, spherical aberration can be mostly eliminated by choosing a microscope objective that matches the refractive index of the tissue. However, in heterogenous structures such as the skin, effective matching is very difficult and significant aberration remains, even when using objectives equipped with correction collars.^25^

Another limitation imposed by tissue physiology on multiphoton skin imaging lies in possible photodamage. While multiphoton microscopy generates less photodamage in thick tissue as compared with most one-photon modalities such as confocal fluorescence microscopy, at the focal volume where photochemical interactions occur, multiphoton processes can still cause considerable photodamage. Today, three multiphoton photodamage mechanisms are well recognized. (a) Oxidative photo-damage can be caused by two or higher photon excitation of endogenous and exogenous fluorophores, with a photodamage pathway similar to that of ultraviolet irradiation. These fluorophores act as photosensitizers in photo-oxidative processes.^26,27^ Photo-activation of these fluorophores results in the formation of reactive oxygen species (ROS) which trigger the subsequent biochemical damage cascade in cells. Current studies found that the degree of photodamage follows a quadratic dependence on excitation power, indicating that two-photon processes are the primary damage mechanism.^28–32^ Flavin-containing oxidases have been identified as one of the primary endogenous targets for photodamage.^28^ (b) Photodamage may also be caused by mechanisms resulting from the high peak power of the fem to second laser pulses. There are indications that dielectric breakdown occasionally occurs.^30^ (c) Most importantly, one-and two-photon absorption of high-power infrared radiation may also produce thermal damage. The temperature change resulting from two-photon absorption has been estimated to be on the order of 1 mK for typical excitation power, and has been shown to be insignificant.^33,34^ However, in the presence of a strong infrared absorber such as melanin,^35,36^ there can be appreciable heating due to one-photon absorption. Thermal damage has been observed in the basal layer of human skin when irradiated by lasers with high average excitation powers.^37^ Masters *et al*. subsequently performed an in-depth study, firmly establishing one-photon absorption by melanin as the primary photodamage mechanism that limits the maximum power that can be used for skin imaging.^38^

### 2.2. The development of multiphoton technology for skin imaging

The demonstration of noninvasive imaging of living embryos by Denk and co-workers firmly established multiphoton microscopy as the method of choice for the high-resolution study of optically thick specimens.^39^ Piston and co-workers performed the first multiphoton *ex vivo* tissue study by imaging cornea structures of rabbit cornea.^40^ Given the optical accessibility of the skin, it is not surprising that the second application of multiphoton microscopy in tissue focused on the skin.^2^ In this study, Masters and co-workers demonstrated that multiphoton microscopy can image the skin down to a pen­etration depth of 150 *μ*m, resolving all the stratified cellular layers in the epidermis and partly into the dermis. In the stratum spinosum, granulosum, and the basal layer, living keratinocytes were observed; the cytosol and the mitochondrial structures were imaged using NAD(P)H fluorescence. Cellular nuclei can be seen as circular voids where fluorescent species are mostly absent. Morphological changes of keratinocytes from a cuboidal geometry (in the basal layer) to a flattened geometry (in the stratum spinosum) were observed, consistent with previous histological studies and confocal microscopy. A bright fluorescent signal was also observed in the *stratum corneum* that may be assigned to keratin today but was not identified in the work of Masters and co-workers. The reteridge morphology of the epidermal-dermal junction was also reconfirmed in this study. In the dermis, a signal from extracellular matrix was also clearly observed, showing the characteristic fibrous structure. Finally, it is important to note that this study was also the first *in vivo* human application of multiphoton microscopy.

Multiphoton microscopy not only allows the imaging of skin morphology in 3D with sub-micron resolution, but the incorporation of spectroscopic measurement further allows quantification of the tissue biochemical state. Excitation/emission spectroscopy and lifetime resolved spectroscopy are two of the most widely used spectroscopic modalities for skin characterization. The utility of both spectroscopic techniques for skin studies were both first demonstrated by Masters and co-workers in the initial multiphoton study of skin.^2^ In this study, emission spectra and lifetime decay kinetics were measured at selected points in the skin, but no spectrally resolved imaging was performed. In the epidermis, the emission spectral measurements and the fluorescence-lifetime resolved measurements both established that NAD(P)H is primarily responsible for the fluorescent signals from living keratinocytes, when excited at approximately 800 nm. In the dermis, the emission spectra show characteristic sharp emission peaks of SHG superimposed on a broad background fluorescence, that today are well recognized as the SHG from collagen and the endogenous fluorescence from both collagen and elastin, but Masters and co-workers did not make the correct spectral assignments in this early study. Subsequently, Laiho and co-workers extended excitation/emission spectroscopy studies in the skin from point measurements to 3D-resolved imaging at several excitation and emission wave-lengths.^41^ They further applied chemometric analysis in order to identify the principal components responsible for the skin endogenous fluorescent signal, resolving contributions from NAD(P)H, collagen and elastin that are well accepted today. They also assigned components corresponding to tryptophan and melanin that are less well supported today. With further instrument improvement, Radosevich and co-workers extended excitation/emission spectroscopic measurement to demonstrate that up to eight different luminescence components can be resolved in the skin.^42^ The eight components correspond to fluorescence signals from NAD(P)H, collagen, elastin, keratin, sebum and flavin protein, combined with second harmonic signals originating from collagen and keratin. Subsequently, excitation/ emission spectrally resolved multiphoton imaging has been applied in a broad range of skin physiology studies.^43–47^ After Masters *et al*. demonstrated life-time-resolved spectroscopy at selected points in the skin, multiphoton microscopes with the capability for lifetime-resolved measurements also saw rapid development. Konig and Riemann demonstrated the first lifetime-resolved imaging of the skin, based on a time-correlated single photon counting (TCSPC) approach.^48^ In conjunction with the development of low-cost, easy-to-use TCSPC modules that can be readily integrated into multiphoton microscopes,^49^ the work of Konig *et al*. established TCSPC as the method of choice for lifetime-resolved skin imaging. Today, the acquisition of 3D multiphoton image stacks of the skin with lifetime-resolved spectroscopic information at every voxel has become feasible, allowing very sensitive discrimination of tissue physiological and pathological states.^11,50–52^

Other nonlinear optical modalities have also found unique applications in dermal studies. Coherent anti-Stokes Raman scattering (CARS) has been shown to be a powerful method to monitor the dermal transport of small drug molecules,^53^ while imaging based on time-resolved transient pump-probe absorption microscopy allows the detection and discrimination of different melanin species.^21,54^ The combination of multiphoton microscopy with other imaging modalities, such as confocal microscopy,^55^ optical coherence tomography,^56,57^ and ultrasound imaging,^52^ have also been shown to be very useful in the study of skin.

## 3. Applications of Multiphoton Microscopy in Different Areas of Dermatological Studies

### 3.1. Skin cancers

A major motivation for the development of multi-photon imaging in skin is the diagnosis and monitoring of skin cancer. The major types of skin cancer by decreasing levels of incidence are basal cell carcinoma (originating from cells that make up the *stratum basale* of the epidermis), squamous cell carcinoma (originating from squamous cells, which make up the major part of the epidermis) and malignant melanoma (originating from melanocytes).^58^ Despite the comparatively low proportion of malignant melanoma cases, it is by far the most deadly form of skin cancer, with a case mortality rate of approximately 20% in the United States.^59^ Multiphoton imaging is particularly applicable for diagnosing this type of cancer, since the identification of abnormally located melanocytes is facilitated by the dark melanin pigment that they produce, unlike basal or squamous cell carcinomas which are much less readily located.

Early examples of multiphoton imaging for skin cancer include a brief mention by Teuchner *et al*., who built a multiphoton microscope and used it to measure the two-photon fluorescence of melanin in excised skin tissue.^60,61^ This was followed later by animal model studies such as in Skala *et al*.,^62^ where a hamster cheek model was used. Tumors were biopsied, imaged in 3D using a titanium-sapphire laser at 780 nm, and five features were determined that could distinguish normal, precancerous and cancerous (squamous cell carcinoma) tissue; this work was later extended by including fluorescence lifetime measurements to quantify the concentration of NADH,^63^ noting that the concentration of protein-bound NADH and FAD was different in tumors when compared to normal tissue.^64^ The use of mouse models of carcinoma is still relevant today; a more convenient xenograft mouse model has recently been developed in order to reduce the need for clinical samples of melanoma tissue.^65^ For skin cancer animal models, dedicated imaging stations have been created in order to perform long-term imaging on mouse carcinoma models.^66^ Time-lapse imaging of tumor-specific CD8 T-cells tagged with green fluorescent protein was performed *in vivo* using two-photon fluorescence. In order to ensure that the same location was imaged over a period of several days, microtattoos were used to facilitate image registration.

While the use of animal models is helpful, the study of human tissues is an important step toward eventual clinical applications. Conventional instruments use two-photon fluorescence of endogenous fluorophores in order to perform imaging. This technique was performed on a tumor biopsy taken from a patient with basal cell carcinoma by Cicchi *et al*.^67^; an increase in fluorescence intensity was observed in cancerous tissue, although it should be stressed that there was only one patient in the study. Another early study imaging malignant melanoma in nevi, used fluorescence with excitation at 760 nm for NAD(P)H, elastin and pigmented cells, followed by excitation at 840nm to highlight pigmented cells and collagen. Melanoma cells were observed to fluoresce much more brightly than surrounding cells.^68^ A much larger trial, with 250 patients, was performed, also imaging melanoma.^69^ The increased fluorescence from cancerous melanocytes was confirmed, and morphological differences could also be seen. A study by Zhang *et al*. further confirmed that the morphology of cancerous melanocytes was different; the cells were more elongated than normal, and the melanocytes appeared to be migrating together. While only one patient was in the study, this marked the first time that this had been observed *in vivo*.^70^ *En face* geometry was also used to image nonmelanoma skin cancer by Paoli *et al*.^71^ and Ericson *et al*.,^72^ who noted that morphological features reported in histopathology could also be observed using multiphoton imaging. In another large study, 115 patients were recruited to study the sensitivity and specificity of two-photon fluorescence for imaging melanoma^73^; values of up to 95% sensitivity and up to 97% specificity were reported. The overarching message of these reports seems to be that while multiphoton fluorescence intensity imaging is suitable for cancer diagnosis, like in histopathology, the morphology is still very important; fluorescence intensity does not serve to highlight cancer cells with enough specificity to be useful.

A potential solution to the problem of not being able to distinguish tumor cells from normal cells is to provide additional contrast mechanisms. Despite the comparatively large number of papers employing fluorescence intensity, it is not the only contrast mechanism that can be used in tissue; fluorescence lifetime imaging^54,74–76^ and spectrally resolved *en face* two-photon fluorescence.^77^ have also been performed, both of which promise better classification of tissue samples. Multiphoton tomography and lifetime imaging were also used to image nevi in an attempt to diagnose malignant melanoma,^78^ and a 37-patient study on the use of spectrally resolved fluorescence lifetime imaging to diagnose melanoma concluded that while different melanized cell types (such as keratinocytes or melanocytes) could be readily distinguished using a single measurement point, morphological data was still necessary to distinguish benign from malignant melanocytic skin lesions.^79^ Morphological differences in basal cell carcinoma and squamous cell carcinoma cells were also observed by Xiong *et al*. using SHG and two-photon fluorescence.^80^ Because SHG is sensitive to type-I collagen, on account of the fact that type-I collagen is one of the few skin components to exhibit noncentrosymmetry, it can be used to distinguish ordered collagen (observed in healthy skin) from disordered collagen which is more common in tumor regions. These two techniques were also used by Chernyavskiy *et al*.^81^ who combined them with confocal reflectance and fluorescence imaging to image the effects of microwave-induced hyperthermia in mice as a treatment for melanoma.

Several other new contrast mechanisms for skin cancer diagnosis are currently in the early stages of development, and their clinical utility remains uncertain. Excited state absorption has been shown to effectively image melanin, and this was developed into a portable imaging system for the diagnosis of melanoma by Teuchner *et al*.^82^ Pump-probe optical coherence microscopy was used to image melanoma by Wan and Applegate,^83,85^ and melanoma was also studied using two-photon photoacoustic microscopy by Oh *et al*.,^84^ who exploited the fact that melanin has a high two-photon absorption cross section. CARS has been combined with SGH and two-photon fluorescence by Vogler *et al*. in order to image basal cell carcinoma.^85^ Opposing the trend of increasing the number of imaging modes, Chen *et al*. limited themselves to just second- and third-harmonic generation using a 1230-nm Cr:Forsterite laser in order to image many different skin features and diseases; they claim that their approach permits greater tissue penetration depth than lasers with shorter wavelengths.^86^

One new trend in melanoma imaging stresses the importance of quantifying the proportion of pheomelanin and eumelanin, since it has been argued that higher proportions of pheomelanin in skin are positively correlated with skin cancer.^87^ This has the potential to be the first case where it is possible to distinguish cancer cells based on spectroscopy alone, rather than having to interpret the morphological data. Fu *et al*. initially built an instrument,^88^ and used transient (or excited state) absorption measurements to distinguish between the two types of melanin in hair, skin and in phantoms consisting of capillary tubes filled with red hair, black hair or Rhodamine 6G.^89^ It was discovered that the excited-state lifetime for eumelanin was much longer, permitting the two species to be imaged separately. This was exploited by Matthews *et al*. to image pigmented lesions that were excised during biopsy.^21^ Contrary to expectation, it was found that there was significantly increased eumelanin in the melanoma samples, and that this fact (combined with other morphological observations) could be used to identify all the melanomas while excluding 75% of the dysplastic nevi, albeit with a sample size of only 21 patients. Further studies have been performed using mouse models,^90^ as well as a study in humans that attempted to image melanogenesis on excised tissue samples.^91^ Fluorescence lifetime has also been used to separate melanoma from nevi,^92^ and attempts have also been made to use ordinary two-photon fluorescence^93,94^ along with a demonstration of the use of two-spectral-channel lifetime imaging to classify nevi and basal cell carcinoma.^51^ Today, while the identification of melanoma based on the spectral differences between pheomelanin and eumelanin is far from proven, this approach does show enough promise to justify further investigation.

While it is fair to say that the majority of multiphoton microscopy cancer research is focused on melanoma, other types of cancer are investigated as well. Cicchi *et al*. used multiphoton imaging to image a number of conditions; besides melanoma, they studied basal cell carcinoma, scarring and keloid formation.^95,96^ More recently, a number of studies on the diagnosis of basal cell carcinoma in excised tissue were performed by groups affliated with König, employing multiphoton tomography,^97^ a combination of multiphoton tomography and fluorescence lifetime,^98^ just fluorescence lifetime,^50^ and a combination of multiphoton and optical coherence tomography,^99^ as well as comparing multiphoton tomography and confocal reflectance for the diagnosis of basal cell carcinoma.^100,101^ Aside from more conventional skin cancers, Hoeller *et al*. offered a unique investigation into T-cell lymphoma, a form of cancer characterized by the accumulation of malignant CD4+ T-cells in the skin.^102^ The investigation was performed by imaging fluorescently labeled malignant T-cells in a mouse model, and several conclusions were drawn as to the biological methods by which malignant T-cells adhere to E-selectin in the skin.

All the papers cited above use some form of endogenous contrast agent in order to perform imaging. The use of endogenous contrast agents for the diagnosis of skin cancer is highly desirable from a complexity, safety, cost, ubiquity and applicability point of view, but exogenous contrast agents may provide spectral contrast sufficient to highlight disease features that cannot be observed using endogenous signals alone. 5-Aminolevulinic acid (ALA) was used by Cicchi *et al*. during a larger study on the imaging of basal cell carcinoma,^103,104^ as well as by Riemann *et al*.^105^; ALA is a precursor to protoporphyrin-IX which is highly fluorescent and accumulates in tumor cells. Gold nanorods are also being pursued on account of their strong luminescence and biocompatibility; Durr *et al*. have spearheaded this research,^106–108^ which has also been pursued by the group of Tunnell.^109,110^ The use of exogenous contrast agents may push the sensitivity and specificity of multiphoton imaging to a point where it can be relied upon in the clinic; however, the regulatory hurdles to the use of any exogenous image contrast agent are considerable.

While conventional histopathology remains the gold standard of any clinical diagnosis including skin cancer, the accuracy of histopathology depends greatly on the skill of the pathologist. A number of research groups are working toward making pathological analysis more quantitative. In the diagnosis of skin lesions based on multiphoton imaging, several research groups advocate the creation of simple, sensitive and robust diagnosis measures. The multifluorescence to SHG index (MFSI) has been proposed as a means of distinguishing basal cell carcinoma from normal tissue,^111,112^ and was used to locate precancerous melanocytes in mice.^113^ The autofluorescence to SHG index (ASI) has also been proposed, and was tested on dorsal skin-fold chambers in nude mice.^114^ Levitt *et al*. developed a much more complex series of image processing metrics for two-photon fluorescence that were used on a tissue model.^115^ Unfortunately, as with many of these studies, only very few of them perform well enough to warrant a large clinical trial in order to evaluate whether they can provide a sensitivity and specificity comparable to, or above that of a trained pathologist.

**Fig. 1:**
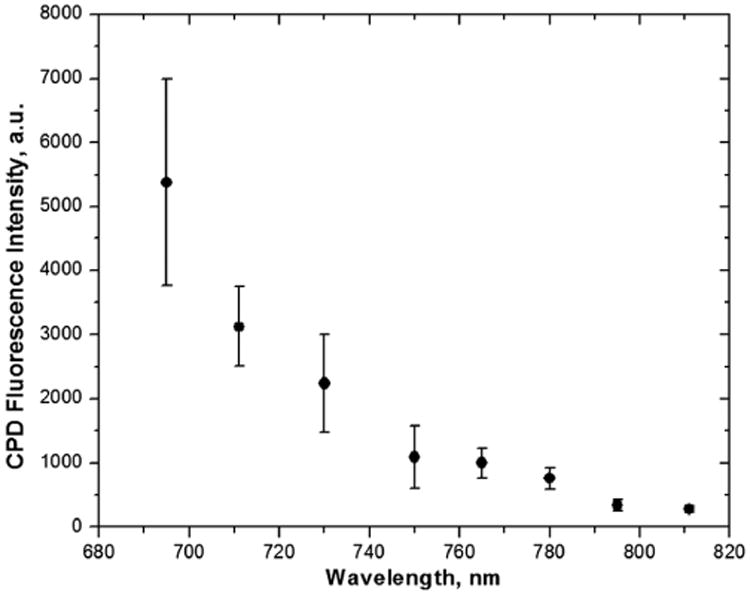
Spectral dependence of CPD damage production. Pixel dwell time: 30 *μ*s, pulse width at the focal plane: 164 fs. Reproduced with permission from Ref. 117.

Two studies published recently highlight possible limitations to multiphoton microscopy or treatment protocols. Kantere *et al*. have noted that multiphoton protoporphyrin-IX fluorescence does not increase tumor contrast; they recommend one-photon anti-Stokes fluorescence instead.^116^ Nadiarnykh *et al*. have an even more striking conclusion; that with a diffraction-limited focal spot and a peak power of around 1 kW, significant DNA damage can occur by multiphoton absorption, as measured by the presence of cyclobutane–pyrimidine dimers.^117^ The effect was strongly dependent on wavelength, with 695-nm excitation being particularly damaging, and wavelengths longer than 780nm being less so (see Fig. 1). This is broadly consistent with the results of Le Harzic *et al*., who noted that 1064-nm femtosecond laser pulses were much less damaging than wavelengths of 532nm and shorter,^118^ and has clear implications for the clinical use of multiphoton imaging in cancer diagnosis.

Overall, the use of multiphoton imaging methods for cancer diagnosis and monitoring shows considerable promise. Many studies have demonstrated that tumors can be identified through a variety of different contrast mechanisms, and while the small field of view precludes the use of multiphoton imaging for wide area screening, it may find use in the investigation of suspicious nevi or other neoplasms, especially in locations such as the head and neck, where the consequences of prophylactic surgical excision are more pronounced. In addition, multiphoton imaging may also find use in margin determination, where surgeons need effective imaging tools to determine whether they have completely resected the whole lesion. The availability of clinically compatible instruments from Jenlab goes a long way in proving that these techniques can be applied not just in a laboratory environment, but work acceptably well in the clinic, and the increasing number of clinical trials that involve a substantial number of patients should provide a statistically significant evaluation of the utility of multiphoton imaging as a clinically useful tool for skin cancer diagnosis.

### 3.2. Other dermatological diseases

In addition to cancer, multiphoton imaging has been used in the diagnosis and monitoring of many different skin diseases and conditions. In the clinic, it has been used to image diseases like Jadassohn–Pellizzari anetoderma,^119^ scleroderma,^120^ lymphedema,^121^ atopic dermatitis in a mouse model,^122^ and actinic keratosis.^123^ In some of these diseases, it is often the fact that both collagen and elastin can be easily resolved by SHG and two-photon autofluorescence, respectively, that makes multiphoton imaging particularly suitable. Jadassohn–Pellizzari anetoderma is characterized by a loss of dermal elastin, whereas scleroderma is characterized by the abnormal accumulation of collagen. Similarly, one of the most important clinical parameters for establishing the extent of lymphedema can be the extent of collagen restructuring. Atopic dermatitis can result in hyperkeratosis (a thickening of the *stratum corneum*) and fibrosis of the upper dermis, both of which can be successfully imaged. Huck *et al*. have pursued atopic dermatitis further, incorporating fluorescence lifetime imaging to measure the proportion of free versus proteinbound NADH as a measure of cellular activity in 20 patients and 20 control subjects.^5,124^ Actinic keratosis has been imaged *in vivo*, with the increased average nuclear diameter being observable both in histopathology and two-photon fluorescence.^123^ Larger studies have been performed by König and coworkers, who imaged a variety of different diseases such as seborrheic keratoses, angioma, actinic keratoses, psoriasis, pemphigus vulgaris, scarring, and autoimmune bullous skin diseases.^125,126^ Skin disease due to infectious agents has also been monitored by multiphoton imaging. Lin *et al*. noted that fungal infections can be monitored by two-photon fluorescence; *Microsporum canis*, for example, is highly autofluorescent and can be readily distinguished from the *stratum corneum* in a mouse model.^127^

Systematic disorders can sometimes also be studied by how they alter dermal structures. Dong and coworkers have studied diabetes. They observed protein glycation, thought to be a major cause of complications caused by diabetes mellitus and aging, by the increased autofluorescence due to the advanced glycation endproducts (AGEs) and slight reduction in SHG in the skin, as well as in the cornea and aorta.^128,129^ Gunawardana *et al*. report on the potential for treating typeI diabetes using tissue engineering: In a mouse model, embryonic pancreatic tissue was implanted subcutaneously,^130^ and two-photon fluorescence imaging used to monitor proper endocrine differentiation.

### 3.3. Skin aging studies

Aside from disease diagnosis and treatment, the cosmetic and plastic surgery industries have considerable interest in multiphoton imaging for the investigation of chronological aging and extrinsic (often photo induced) aging of skin. Lin *et al*. first attempted to create a measure that correlated with the chronological age of a subject; the SHG to autofluorescence aging index of dermis (SAAID) offered a simple means to estimate the age of skin by taking the ratio of SHG to autofluorescence.^131,132^ This measure has been supported by a study which noted that there was a difference in SAAID scores between men and women of the same age,^133^ and another study which confirmed the correlation with age by measuring sites on the face.^134^ The discrepancy between men and women was overcome by a more subjective score, the MLT-based dermis morphology score (MDMS, where MLT stands for multiphoton laser-scanning tomography) which also had a better correlation with age than SAAID.^135^ Depth-resolved measurements of SAAID were taken by Kaatz *et al*., in order to quantify to what extent the measure varied with imaging depth.^136^ The difficulty in using SAAID to distinguish between photoaged and nonphotoaged skin was noted by Sanchez *et al*. and an improvement, incorporating lifetime imaging of NAD(P)H, was made.^137^ Life-time imaging was also used by Koehler *et al*. to diagnose dermal elastosis, which is often a sign of extrinsic aging.^138^

Other researchers have proposed similar measures; Puschmann *et al*. proposed the elastin to collagen ratio (ELCOR) which explicitly includes autofluorescence contributions from just elastin by manual masking of the image, and also takes the ratio of the area of each skin component as opposed to the fluorescent intensity.^139^ Wu *et al*. proposed a measure based on the fast Fourier transform of the SHG image^140^ or the gray-level cooccurrence matrix.^141^ Cicchi *et al*. have also proposed a similar method based on the fast Fourier transform.^142^

Other studies were more qualitative, seeking to investigate the changes that occur during chronological aging or photoaging. Koehler *et al*. have investigated the acceleration of aging induced by sunbeds; while the sample was too small to quantify the damage from the sunbed, differences were observed between the dorsal and volar forearm, demonstrating the effect that sun exposure has on skin.^143^ Similar observations were made by Benati *et al*.^144^ and Baldeweck *et al*. who performed 3D-resolved measurements to further investigate the differences between exposed and unexposed skin.^145^ Decenciere *et al*. extended this 3D resolution further, developing a segmentation algorithm which could be used to quantify the size and shape of various skin components, and in the process, estimate the effect of aging.^146^ yasui *et al*. used polarization-resolved SHG to show that wrinkles in skin were aligned with the underlying collagen fibers,^147,148^ and Lutz *et al*. argued that collagen cross-linking could be quantified *in vitro* by noting the increase in SHG and a decrease in the fluorescence lifetime.^149,150^

**Fig. 2:**
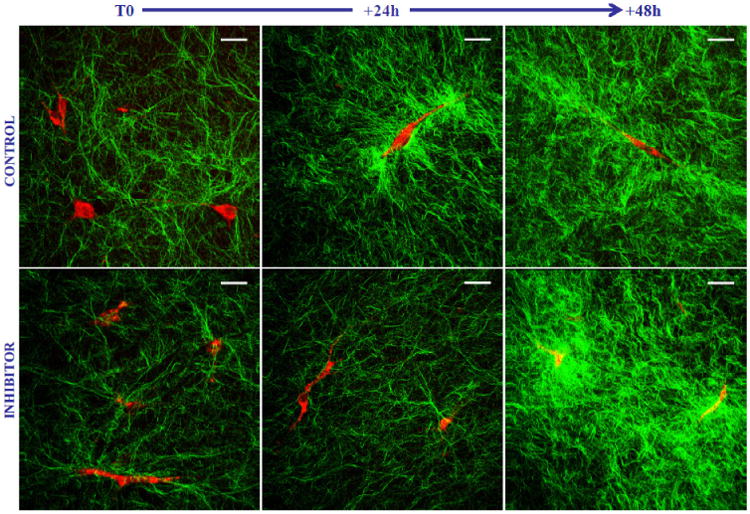
Multiphoton imaging of collagen matrix remodeling induced by fibroblast contraction. Combined 2PEF (red) and SHG (green) images of fibroblasts within control and Y-27632 treated collagen gels. The images were acquired at T0-before samples contraction; T+24 h and T+48 h — 24 h and 48 h after free contraction of the samples. At T+24 h, the fibroblast inhibitor was removed from the culture medium and replaced with a control culture medium in order to assess the reversibility of the inhibitor effect. Scale bar: 30 m. Excitation: 60 mW at 730 nm. Objective: 20×, 0.9 NA. Acquisition time: 6.9s/image of 681681 pixels. Reproduced with permission from Ref. 152.

The therapeutic effects of certain skin treatments have also been investigated using multiphoton imaging. Pena *et al*. investigated a potential treatment for wrinkles, by noting that fibroblasts can cause contraction in the skin. The effect of Y-27632, a RhoA-kinase inhibitor, was investigated, and found to have potential as a means to inhibit this contraction^151,152^ as seen in Fig. 2. Bazin *et al*. also investigated a potential anti-wrinkle treatment consisting of soy and jasmine extracts, and showed a statistically significant increase in dermal collagen content.

Aside from biochemical agents, the after effects of laser treatment for wrinkles have been investigated using two-photon fluorescence and SHG; laser fractional micro-ablative rejuvenation involves using a laser to induce a thermal shock to fibroblasts in the skin, which then produce more collagen. This increased collagen production can be imaged using multiphoton microscopy.^153–155^ A very similar study was previously performed by Tsai *et al*., investigating the effect of Er:YAG laser irradiation on skin for the treatment of skin hyperplasia and tumors.^156^

### 3.4. Skin regeneration studies

Wound healing and dermal regeneration processes have also been studied with multiphoton imaging, since type I collagen can be imaged particularly well using SHG imaging. As the structure and morphology of the collagen that forms around the wound plays a large part in determining whether a scar forms, or whether the scar is normal, atrophic, hypertrophic or keloid, this application is particularly suited to SHG imaging. Initial studies focused on observing wound healing process in animal models. Navarro et al. imaged the different stages of skin wound closure in guinea pig models using two-photon fluorescence at several time points after full-thickness wounds were induced surgically. Growth of blood vessels and collagen fibers was observed.^157^ This was pursued further, incorporating SHG imaging, and imaging the wound with greater time resolution and over a longer period, in order to yield more insight into the wound-healing process.^93^ Later, Luo *et al*. used SHG and image analysis to investigate wound healing in KunMing mice over a period of 14 days.^158^

Human scarring has been studied with *ex vivo* specimens. Brewer *et al*. imaged samples excised from normal and keloid scars. While the study was very limited (with a total of two patients), a difference in collagen density was observed, although the trend was contrary to expectations, with a greater collagen density observed in the normal scar as opposed to the keloid one.^159^ Meshkinpour *et al*. used SHG to image biopsies taken from keloid and hypertrophic scars undergoing treatment with the ThermaCool (TC) device from Thermage Inc. Significant variation was found in the collagen structure of the four biopsied patients.^160^ This finding was echoed by Da Costa *et al*. who found swirling collagen structure in keloids, as opposed to more wavy structure in normal skin.^161^

*In vivo* study of human scarring was first studied by Riemann *et al*., imaging a biopsy scar of a single patient over a period of 60 days, with images taken every one to three days after surgery. Two-photon fluorescence and SHG were both employed, and the organization of the new collagen fibers was noted.^162^ Zhu *et al*. imaged much older scars by sampling from women who had previously undergone caesarian section. Their data showed a slight decrease in elastin fluorescence and SHG from collagen as the wound aged.^163^

**Fig. 3:**
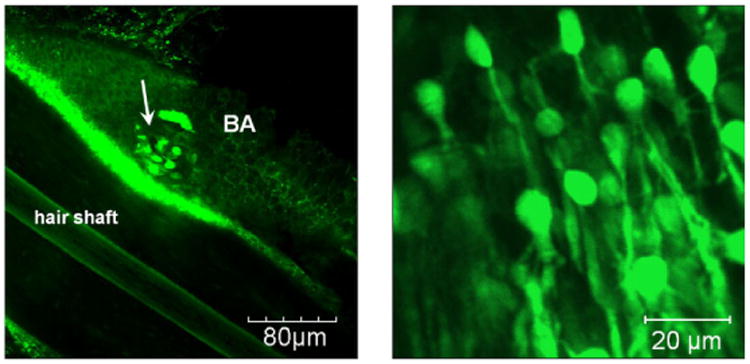
Typical nestin-GFP expressing stem cells within the hair follicle bulge (white arrow). The cells have an oval-shaped body with a typical size of 7mm and dendritic-like arms. Images were obtained from fresh isolated hair follicles by confocal 3D optical slicing. Reproduced with permission from Ref. 166.

The skin is known to possess adult stem cells, specifically within the hair follicles. These stem cells may be found in the bulge area as well as in the dermal papilla. Using two-photon microscopy, Rompolas *et al*. studied the growth regulation of these stem cells *in vivo* in mice.^164^ Through the observation of hair follicle regeneration, they found that there exists a spatial organization to these stem cell progeny divisions. Likewise, cell-to-cell signaling allows for coordinated and rapid movement of the follicle. Through targeted laser ablation, Rompolas *et al*. also showed that the mesenchyme plays an important role in hair regeneration. Similarly, Liu *et al*. studied the pluripotency of the nestin expressing stem cells found within the bulge area and the dermal papilla.^165^ These cells migrate from the bulge area to the dermal papilla, suggesting that the bulge area is the source of skin stem cells^166^ as seen in Fig. 3. By seeding these cells on Gelfoam and subsequently transplanting them into mice with spinal cord injury it was observed that the transplanted cells migrated toward adjacent spinal cord segments. Mice that were transplanted with these stem cells experienced plantar placing of the affected paw within three days of transplantation whereas the negative control group transplanted with only Gelfoam took seven days. Full recovery took at least 28 days for the mice transplanted with stem cells, while only a few mice in the untransplanted group achieved locomotor recovery.

Several researchers have noted that the ratio of two-photon fluorescence to SHG can be used to classify scars^167,168^; however, as collagen structure is deemed to be important in wound healing, several researchers have attempted to quantify the degree of order within a collagen structure. Chen *et al*. contrasted three different approaches; an edge-detecting filter to determine the gradient at each pixel, a simple threshold to measure the collagen density for the image and a complex semi-arbitrary geometric morphology approach. These were then weighted to form a final measure.^169^

Some collagen classification metrics that are discussed in the skin aging section, also find applications in wound healing. Cicchi *et al*. also assessed three different approaches, including employing a gray-level co-occurrence matrix (which was studied further by Ferro *et al*.^170^), a fast Fourier transform and a measure known as the SAAID, or Second-harmonic Autofluorescence Aging Index of Dermis.^142^ Rather than determining one superior technique, it was found that each was effective at a particular length scale. Jiang *et al*. used a fast Fourier transform as well, in order to define a Collagen Orientation Index and a Bundle Distance, which was then used to characterize collagen in the deep, middle and superficial dermis of keloid tissue excised from patients undergoing reconstructive surgery.^171^ Taking the above approaches further, it is possible that image processing combined with multiphoton imaging can help determine the border of a scar, to aid the surgeon in determining where to excise or intervene. Chen *et al*. used two-photon fluorescence and SHG to image skin samples taken from six patients, five of whom had hypertrophic scars. After processing the images, a number of features were proposed in order to distinguish scar tissue from normal tissue.^172^ Shortly afterward, another set of researchers discovered that the volume density of elastin could be used to distinguish keloid, hypertrophic and normal scars,^173^ and that the two-photon fluorescence and SHG from collagen could be used to distinguish atrophic and keloid scars.^174^

In terms of instrument development, Su *et al*. published two papers demonstrating polarization-resolved SHG, and showed that the second-order susceptibility ratio *d*_33_=*d*_31_ could be used to distinguish normal tissue from keloid, morphea and dermal elastolysis.^175,176^ The most significant developments in applying multiphoton imaging to patients *in vivo* have been made primarily by König and co-workers; in particular, the use of a GRIN lens to allow imaging from within atrophic scars and other recessed skin features, and to image ulcers *in vivo*.^11,177–179^ Later, a GRIN lens with a higher numerical aperture of 0.8 was introduced, providing increased spatial resolution.^180^

### 3.5. Transdermal transport of drugs, cosmetics, sunscreens and nano-particles

The skin forms a natural barrier that protects the body by keeping potentially toxic substances out. This barrier comprises of a physical layer (the *stratum corneum),* as well as immunological and enzymatic defenses.^181^ Many cosmetic and pharmacological products are designed and sold for topical application, and their efficacy sometimes depends on their penetration into the interior of the skin. The dermal distribution of these products has been studied, and much work has gone into designing formulations that can effectively overcome the skin barrier.^182^

Grewal and co-workers were the first to demonstrate that penetrant distribution in skin can be noninvasively visualized using multiphoton microscopy.^183^ They further demonstrated that the fluorescently labeled dextran distribution can be modulated by topical application of different enhancers. Subsequently, Yu *et al.* determined the distribution of fluorescent hydrophobic and hydrophilic penetrants in the skin both before and after treatment with oleic acid, a common enhancer. Coupled with biochemical diffusion rate measurement data, they first quantitatively extracted transport parameters such as concentration gradient enhancement factor and the probe vehicle to skin partition-coefficient enhancement factor . They further proved that hydrophobic and hydrophilic agents penetrate through the skin *stratum corneum* and the epidermis through different routes.^184^ Yu and co-workers further showed that, due to skin heterogeneity, high throughput large area multiphoton imaging is critical to minimize errors in determining penetrant transport properties.^185^ Finally, they have also quantitatively evaluated the effect of ultrasound in skin transport enhancement.^186^

Besides transport, the safety of topically applied cosmetics, lotions and creams is of key importance for the cosmetic and drug industry. Furthermore, with the development of nanoparticles for a variety of applications, the biosafety of these particles due to inadvertent absorption through skin is also an important concern. For example, nanoparticles such as zinc oxide or titanium oxide are between 20 and 30 nm in size and are often used in sunscreens.^18^ Using two-photon microscopy on excised human skin from volunteers, it was found that these nanoparticles remained in the *stratum corneum.* Higher concentrations were found in the skin folds or hair follicle roots; on the order of 800 particles/ *μ*m^3 .188,189^ Pigment particles remaining in the skin after tattooing represent another class of common nanoparticles in human skin. Konig investigated how nanoparticles from tattoo pigments could be imaged, showing that they could be distinguished from other autofluorescent species in the skin by their fluorescence lifetime and emission spectrum.^190^ Efforts have also been made to devise a multi­dimensional quantitative approach toward skin penetration by pharmacological formulations. Recently, a multimodal approach utilizing multi-photon imaging has seen early clinical trials as a way of performing optical biopsies, as well as testing the efficacy of cosmetics.^192,193^ In addition, Saar and co-workers used stimulated Raman scattering (SRS) to investigate, noninvasively and label-free, the penetration of drugs into the skin. Using ibuprofen and ketoprofen in propylene glycol applied to an excised mouse ear, they were able to image a three-dimensional volume (250 × 250 × 100 *μ*m^3^ ) of SRS data, which extended from the applied drug on the surface to the subcutaneous fat. In their study, they found that both drugs penetrated through the intercellular lipids of the *stratum corneum* and the hair shafts. Saar and co-workers were able to use SRS microscopy to track chemical uptake and transport kinetics. They found proof that transport through the *stratum corneum* was slower, taking over 2 h, compared to penetration through the hair shaft, which reached steady-state in 26min. With the development of video-rate SRS microscopy^194,195^ and the label-free capability of SRS, real-time tracking of the efficacy of cosmetic and sunscreen compounds can be imaged with both high spatial and temporal resolution.

**Fig. 4:**
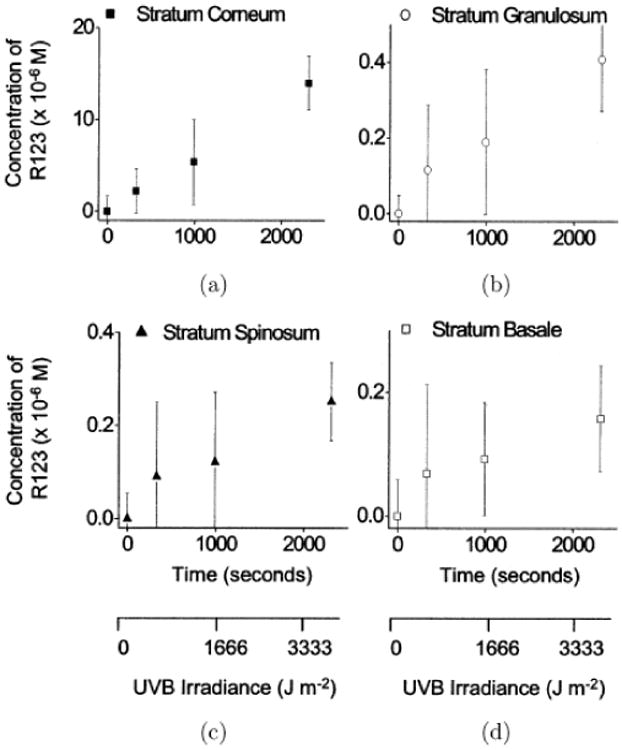
Concentration of R123 as a function of UVB irradiation time (upper x-axes) or UVB dose (Jm^−2^, lower x-axes*)* in the *stratum corneum* (a) and the viable epidermal strata (b-d) for lightly pigmented skin. Variability in the signal most likely results from histological, pigmentation and age difference between samples. Reproduced with permission from Ref. 196.

The importance of being able to study the efficacy of such compounds noninvasively as well as with high resolution is demonstrated by the work by Hanson and Clegg.^196^ In their study, they observed and quantified the generation of ROS in *ex vivo* skin irradiated with UV light. In this study, *ex vivo* human skin samples were incubated with dihy-drorhodamine-123 (DHR), which only converts to a fluorescent form (rhodamine-123) after reacting with ROS. The samples were then irradiated with varying doses of UVB and the amount of rhodamine-123 generated is imaged with a two-photon microscope and quantified. It was found that for an average adult-sized face exposed to 2h to UVB generated 14:7 × 10^−3^ moles of ROS (as measured by their reaction with DHR-123 to form fluorescent rhodamine-123) in the *stratum corneum.* A further 10^−4^ moles were generated in the lower epidermal strata. A subsequent study further revealed that some UV blockers actually increased the amount of ROS generated (see Fig. 4), and therefore increased the chances of skin cancer.^197^ Since pH gradient may affect the transport of polar chemical species, a high resolution study of the *stratum corneum* pH gradient was also performed, and it was found that the acidity decreases with increasing depth.^198^

The effect on the skin of common chemical warfare agents are clearly of importance from the standpoint of protection and treatment. Werrlein *et al.* have investigated the effects of sulfur mustard, a potent vesicant, on human epidermal keratinocyte cultures.^199^ They noted a disruption to actin filaments, large punctuate inclusions and a lack of stress fibers in exposed cells, as compared to controls.

## 4. Conclusion

While skin is one of the most accessible organs in the body, the physiology of the skin is complex and far from being fully understood. The development of powerful imaging tools based on multiphoton techniques enables *in vivo* minimal invasive imaging of skin physiology throughout the epidermis and into a substantial fraction of the dermis. The *in vivo* imaging of stem cells and their physiological functions in the native skin environment by the König group will likely provide significant insights for stem cell technology and regenerative medicine.^171^ Similarly, skin pathologies are medically important. While chronic diseases, such as dermatites, are not life threatening, they can significantly compromise the quality of life for patients. Melanoma, the most dangerous form of skin cancer, can be very effectively cured if the lesion is discovered sufficiently early, but the 10-year mortality rate can be in excess of 70% after metastasis has occurred. Interestingly, a recent report has shown in a mouse model that melanoma may develop from “invisible” nevi that contain difficult-to-visualize lightly colored eumelanin instead of the darker pheomelanin.^22^ The possibility of the presence of melanoma-causing invisible nevi in light skin color population is an important medical hypothesis that should be investigated. Recent multiphoton imaging technologies^21^ that can effectively distinguish eumelanin from pheomelanin can play an important role in these studies, and may play an important future diagnostic role if this hypothesis is validated. Lastly, skin products, used for cosmetic, sun-protection, anti-aging or regeneration purposes, are commercial products that we use daily. Despite their financial significance, the efficacy of many of these products is mostly judged subjectively. Importantly, the toxicology evaluation of many of these products is often phenomenological and sometimes lacking the vigor of modern physiological investigation. The advent of multiphoton microscopy, which enables the *in vivo* study of many of these products on animal models and human volunteers is promising to change the research of this large and commercially important field.

Shortly after the advent of biological multiphoton microscopy with the publication of Denk and co-workers in 1990, the imaging of skin was recognized as an important biomedical area, and one in which this exciting imaging modality will find unique application. Significant technological progress has occurred over the past decade. Multiphoton skin imaging has progressed from using bulky, slow laboratory-based microscopes^2^ to ergonomic, fast, regulatory agency approved clinical devices.^11^ The significance of gaining regulatory approval of this technology and the establishment of the safe operation limit cannot be over-emphasized. This important step has now pushed open the doors of clinics around the world for the multiphoton microscopic investigation of skin physiology and pathology, which will, in turn, probably ease the way for clinical trials using multiphoton microscopy to study the other organs.

While multiphoton skin imaging technology has progressed significantly along many fronts, the format of a review provides a forum for the authors to speculate on the most important future directions in multiphoton skin imaging instrumentation. Though multiphoton imaging has been demonstrated in the brain up to a depth of almost 2 mm, the typical imaging depth in skin is substantially smaller, in the region of 150 to 200 *μ*m. A major direction in technique development must therefore be to image deeper into the skin. In a way, the fact that tissue penetration is superior in the brain is not surprising, given the higher scattering coefficient of the skin compared with the brain.^200^ However, just the difference in scattering coefficients does not appear to fully account for this difference. This difference may be partly explained by the fact that skin imaging is based on endogenous fluorophores such as NAD(P)H, which emit at approximately 450 nm, a wavelength that has short mean free path in tissues. On the other hand, the deep imaging work in the brain is often based on bright exogenous fluorophores emitting more at red wavelengths. The two-photon excitation wavelength of NAD(P)H is 730–800 nm, which is substantially shorter than the deep brain imaging work at 1200 or 1800 nm. This difference may also be partly explained by the fact that deep brain imaging is targeting relatively larger vascular structures, while skin imaging focuses more on detailed features like the morphology of individual cells or that of elastin fibers. Finally, we believe that the difference in penetration depth between the brain and the skin is also partly due to the layered structures of the skin, each with a different refractive index, which result in significant aberration as one images deeper into the tissue. This is a clear opportunity for suitable correction using adaptive optics. While adaptive optics systems have been developed for multiphoton microscopy,^201^ its actual use in *in vivo* clinical imaging is very limited. This is partly because, in the imaging of many biological systems, the advantage of using adaptive optics is relatively modest in most cases.^202,203^ However, it is possible that adaptive optics is perfectly suitable for skin imaging and may substantially improve imaging depth. It would be interesting to label dermal capillaries with red emitting dye and study the depth of penetration in the skin using excitation wavelength in either 1200- or 1800-nm range. The drawback is that, while this approach may work with exogenous organic probes or deep red fluorescent proteins, endogenous contrast agents like NAD(P)H cannot be readily excited. Of course, this provokes the question: Can we identify other endogenous proteins that can provide multiphoton contrast, either based on fluorescence or absorption? The answer is that potentially, the imaging of flavins, cytochromes and porphyrins with longer excitation and absorption wavelengths may result in some improvement in penetration depth, but it remains to be proven.

Another major development that is revolutionizing the whole multiphoton imaging field is the use of newer contrast mechanisms that complement older nonlinear optical contrast mechanisms such as multiphoton fluorescence and SHG. As discussed in this review, techniques based on Raman and absorption contrast hold tremendous promise. Absorption contrast between eumelanin and phomelanin is one of the highlights of recent research, and potentially has significant clinical relevance. Hypothetically, the combination of absorption and fluorescence may allow the study of tissue metabolism with an accuracy and precision that was not previously available.

Metabolic imaging is partly limited by the fact that NAD(P)H is fluorescent while NAD is not; similarly, only oxidized flavins are fluorescent while the reduced forms are not. The paradigm established by Chance and co-worker almost half-a-century ago of ratioing oxidized flavin with NAD(P)H to obtain metabolic imaging was a breakthrough.^19^ However, if one can obtain the direct ratios of NAD versus NAD(P)H and oxidized versus reduced flavin, one may potentially be able to dissect cellular and tissue redox pathways with much greater precision.

The importance of imaging using chemical signature based on Raman imaging has been demonstrated in many fields, and only partly in skin. While there are some successes in the imaging of fluorescent penetrant distributions, the presence of a bulky fluorophore linked to the small molecule of interest greatly compromises the relevance of many of these past studies. Although some drugs and chemicals are intrinsically fluorescent, they are in a minority, so the work by Xie and co-workers in imaging the penetration through skin by small, nonfluorescent molecules, such as retinol and propylene glycol, is a breakthrough.^201,204^ It is likely that Raman-based skin imaging will become the method of choice for imaging drug and chemical transport processes in the skin.

Finally, while it is a significant advance to port a laboratory-scale multiphoton microscope to a robust articulated arm system that is compatible with clinical applications, the ability to drastically miniaturize this system to a hand-held probe or an endomicroscope format will even further enhance the clinical acceptance of multiphoton imaging of skin. It is also important to note that while the system has been significantly improved, the current acquisition speed of the clinical system is not significantly faster than that of the laboratory model, being limited to a frame rate of several Hz in order to achieve a good signal to noise ratio. While there are many high-speed multiphoton imaging approaches being developed that preserve the image signal-to-noise ratio while greatly improving image speed,^205^ most of these techniques, such as selective imaging based on acousto-optical deflectors, and parallelized imaging based on either multi-foci or temporal focused wide field excitation, have not been adapted for clinical imaging. Clearly, the adaptation of these more-advanced multiphoton imaging techniques in a miniaturized endomicroscope format will present a significant challenge.

In this short review, we have discussed the physiology and optical properties of the skin and the historical trajectory of multiphoton technology developments in the imaging of this complex organ. Most importantly, we have provided a fairly thorough review of the major applications areas that use multiphoton microscope for skin imaging, including the diagnosis of skin cancer and other diseases, the assessment of skin aging and the regeneration process, and the monitoring of transdermal transport of different drugs, chemicals and nanoparticles. There is no doubt that, with further technological advances, multiphoton microscopic imaging will become the method of choice for the scientific study of skin physiology and pathology, but if a number of additional challenging clinical, regulatory, economical hurdles can be overcome, it is possible that multiphoton imaging may one day become an important tool in routine patient care as well.
